# MentaLiST – A fast MLST caller for large MLST schemes

**DOI:** 10.1099/mgen.0.000146

**Published:** 2018-01-10

**Authors:** Pedro Feijao, Hua-Ting Yao, Dan Fornika, Jennifer Gardy, William Hsiao, Cedric Chauve, Leonid Chindelevitch

**Affiliations:** ^1^​ School of Computing Science, Simon Fraser University, Vancouver, Canada; ^2^​ École Polytechnique, Université Paris-Saclay, Palaiseau, France; ^3^​ BC Centre for Disease Control, Vancouver, Canada; ^4^​ School of Population and Public Health, University of British Columbia, Vancouver, Canada; ^5^​ Department of Pathology and Laboratory Medicine, University of British Columbia and BC Centre for Disease Control, Vancouver, Canada; ^6^​ Department of Mathematics, Simon Fraser University, Vancouver, Canada

**Keywords:** multi-locus sequence typing, next-generation sequencing, pathogen surveillance

## Abstract

MLST (multi-locus sequence typing) is a classic technique for genotyping bacteria, widely applied for pathogen outbreak surveillance. Traditionally, MLST is based on identifying sequence types from a small number of housekeeping genes. With the increasing availability of whole-genome sequencing data, MLST methods have evolved towards larger typing schemes, based on a few hundred genes [core genome MLST (cgMLST)] to a few thousand genes [whole genome MLST (wgMLST)]. Such large-scale MLST schemes have been shown to provide a finer resolution and are increasingly used in various contexts such as hospital outbreaks or foodborne pathogen outbreaks. This methodological shift raises new computational challenges, especially given the large size of the schemes involved. Very few available MLST callers are currently capable of dealing with large MLST schemes. We introduce MentaLiST, a new MLST caller, based on a k-mer voting algorithm and written in the Julia language, specifically designed and implemented to handle large typing schemes. We test it on real and simulated data to show that MentaLiST is faster than any other available MLST caller while providing the same or better accuracy, and is capable of dealing with MLST schemes with up to thousands of genes while requiring limited computational resources. MentaLiST source code and easy installation instructions using a Conda package are available at https://github.com/WGS-TB/MentaLiST.

## Data Summary

All experimental results have been deposited in a Figshare collection; DOI: https://doi.org/10.6084/m9.figshare.c.3856300.v2 (url https://figshare.com/collections/MentaLiST/3856300)

**Impact Statement**

Our work proposes MentaLiST, the first algorithm for determining multi-locus sequence types (MLST) from whole-genome sequencing data that is fast, memory-efficient, scalable to whole-genome MLST schemes, and requires no preprocessing step such as assembly, read mapping or tree construction. Furthermore, it is robust in situations of low coverage and the presence of minor strains in the sample, making it appropriate even for samples that would be challenging to call with any other method. MentaLiST opens up the possibility of determining the MLST types of hundreds of strains by typing thousands of genes, in a matter of hours. This in turn creates exciting opportunities for further processing (such as outbreak strain clustering, lineage assignment and determination of drug resistance profiles). MentaLiST will be of interest to computational biologists, microbiologists, epidemiologists and public health practitioners.

## Introduction

Since it was introduced by Maiden *et al.* in 1998 [1], multi-locus sequence typing (MLST) has become a fundamental technique for classifying bacterial isolates into strains. It has been applied in a large number of contexts, especially related to pathogen outbreak surveillance [2]. MLST works by associating to an isolate a sequence type defined by a specific allelic profile based on an established MLST scheme. MLST schemes exist for many important pathogens [3].

Prior to the use of whole-genome sequencing (WGS) data, MLST schemes were based on a small number of carefully selected housekeeping genes (usually fewer than 10), which made the approach both portable with respect to first-generation sequencing technologies (mostly Sanger sequencing) and able to accommodate pathogen evolutionary modes that might confound evolutionary analysis, especially lateral gene transfer [2].

Recently however, several case studies illustrated that the reliance on a small MLST scheme might not provide enough resolution to separate isolates into epidemiologically meaningful clusters. For example, Jolley *et al.* showed that traditional MLST schemes were not able to discriminate separate sublineages within a clonal complex of *Neisseria meningitidis* [4]. This observation has come at a time when advances in sequencing technologies and protocols have had a major impact on public health, as it is now common to rapidly obtain WGS data from a pathogen outbreak, allowing for monitoring at an unprecedented level of resolution [5–13]. In the specific case of MLST, this has led to the emergence of MLST schemes based on a larger set of genes, such as *core genome* MLST (cgMLST), that consider the set of core genes shared by a group of related strains (generally a few hundred genes), and even *whole genome* MLST (wgMLST) schemes that rely on a set of thousands of genes, covering most of the loci of the considered isolates [14]. Since their introduction, cgMLST and wgMLST have proven to be valuable typing methods in many studies [15–20] and are expected to become standard approaches for pathogen surveillance [21].

These new developments in pathogen isolate genotyping motivate the development of MLST software able to accurately classify isolates into sequence types from large-scale WGS data that scale well in terms of the computational resources required, in order to handle large MLST schemes. Very few MLST tools that can meet these requirements currently exist. Indeed, most MLST typing programs have been developed with small schemes in mind, and are based on the availability of assembled genome sequences for the isolates being considered, or, if WGS data are provided, require an initial stage of contig assembly prior to the specific genotyping phase [12, 22–27]

This approach suffers from the computational cost of assembling genomes, but more importantly for large MLST schemes, from the fact that reads corresponding to some loci in the scheme might not be assembled into contigs due to depth of coverage or other assembly issues. Other approaches have been developed recently that bypass the need to have assembled genomes or contigs and rely on directly mapping short reads onto the allele database for a given MLST scheme [28, 29]. However, the initial mapping phase is costly, especially for large schemes that can contain tens of thousands of alleles. Lastly, a few recent approaches have tried to avoid costly preprocessing of short read datasets by working on the principle of k-mer indexing, which has been shown to be helpful in handling large short-read datasets in other bioinformatics contexts such as metagenomics [30]. Two tools that follow this approach currently exist: stringMLST [31] and StrainSeeker [32], although the latter assigns isolates to the nodes of a guide-tree which is required prior to the typing phase.

In this paper we introduce MentaLiST, a k-mer based MLST caller designed specifically for handling large MLST schemes. We test MentaLiST on several datasets, including a new cgMLST scheme for *Mycobacterium tuberculosis* composed of 553 essential genes, and compare its performance with that of two recent MLST callers, stringMLST, another k-mer based tool and ARIBA, a recent assembly-based tool. Our tests show that MentaLiST achieves comparable or better accuracy levels than both stringMLST and ARIBA while consistently using a low amount of memory and requiring much less computation time.

## Methods

We propose a method for MLST calling that does not require pre-assembled genomes, working directly with the raw WGS data, and also avoids costly preprocessing steps, such as contig assembly or read mapping onto a reference. MentaLiST uses an algorithm that follows the general principle of k-mer counting, introduced in stringMLST [31], with some data compression improvements that lead to much smaller database sizes and a faster running time.

The general principle is to find all k-mers present on the MLST scheme alleles, for each locus, and store this information as a k-mer hash map in an index file. Then, for each k-mer in the reads of a given sample, all alleles that contain this k-mer will receive one vote. The allele called for each locus is then the one with the most votes. The novelty on MentaLiST is in the construction of a coloured de Bruijn graph [33] for each locus of a given MLST scheme, which allows the selection of a small subset of the MLST scheme k-mers, drastically reducing the size of the index file created by MentaLiST and improving the running time, at no precision cost. Each step of the algorithm is detailed in the following subsections.

### Coloured de Bruijn graph and k-mer hash map

Before calling the alleles for a given sample, a preprocessing step is required, that needs to be executed only once for a given MLST scheme.

The objective of the preprocessing phase is to build a k-mer database for a given MLST scheme and a given value of *k*. In the uncompressed version of the algorithm, for each locus in the scheme, the k-mers of all alleles of the locus are computed, and a hash table linking each k-mer to the alleles where it is present is created. In this hash table, each k-mer points to a list of all the alleles containing this k-mer. The same k-mer can be found in different loci, although the larger the value of *k*, the less likely this is to happen.

**Fig. 1.**
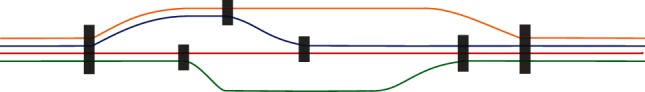
Sketch of a coloured de Bruijn graph with four alleles, each represented by a different colour. The branching nodes are marked in grey, and paths between those nodes correspond to contigs. All nodes of the same contig have the same set of colours.

It is possible to compress this hash map by storing a subset of the k-mers, that still represent all sequence variation seen in the alleles. This is done with the construction of a coloured de Bruijn graph [33] for each locus of a given MLST scheme. A *de Bruijn graph* is a graph whose vertices are the k-mers observed in the alleles of the given locus and whose edges connect k-mers of the form *ax* and *xb*, where *a,b* {A,C,G,T} are single nucleotides and *x* is a common (k−1)-mer. A *coloured* de Bruijn graph has the extra information, on each k-mer node, of which *colours* (alleles) have this k-mer. A *contig* is a path in this graph, starting and ending at branching nodes (i.e. nodes of degree greater than 1), and containing no internal branching node. A pair of contigs with the same starting and ending branching nodes is commonly called a *bubble* and represents sequence variation between different alleles, as seen in Fig. 1.

An interesting property of this graph is that all k-mers on the same contig have the same set of colours. Therefore, instead of storing all *n* k-mers for a particular contig of length *n*, we store only one representative k-mer, set its weight to *n*, and use this weight in the voting phase of the calling algorithm. The intuition for this idea is that, instead of storing *n* redundant k-mers that have the same colours (alleles), we can store only one; its weight of *n* then represents the number of votes that it is entrusted with, and this allows us to store a fraction of the information. Assuming that the whole contig has been sequenced, which is likely to be true given a high enough depth of coverage, the choice of the representative k-mer can be arbitrary, and the resulting vote counts are basically the same as in the uncompressed version, with a slight variation due to non-uniform coverage.

We also applied another compression technique, on the allele list storing. Since most alleles have very similar sequences, with small differences such as single nucleotide variations (SNVs) or small indels, the large majority of k-mers are either present in most alleles, or in just a few (if they overlap a variable position). Therefore, instead of storing the list of all the alleles containing a k-mer, if this list contains more than half of the total number of alleles, its *complement* is stored instead, and the weight of this k-mer is also complemented (multiplied by −1). This means that all the alleles that *do not* have this k-mer get negative votes, which achieves the same net effect as giving a positive vote to all the alleles that do have it. As there are often loci with hundreds to a couple of thousand alleles and MentaLiST is designed to handle MLST schemes with hundreds to thousands of genes, this method significantly decreases the hash map memory footprint, while speeding up the allele calling phase since a lot fewer alleles are likely to be involved in the voting step.

### k-mer counting and voting

For a given sample, MentaLiST iterates through each sequenced read, k-merizing the read and checking each k-mer hit in the k-mer hash map. Each of its k-mer hits gives a weighted vote for all the alleles in which this k-mer is present, as found through the hash map. After all the reads have been processed, the allele with the largest vote count is selected for each locus. In the case of a tie, a random allele is selected among those with the most votes. MentaLiST outputs a log file with the number of votes for each allele in each locus and a list of tied alleles.

**Fig. 2.**
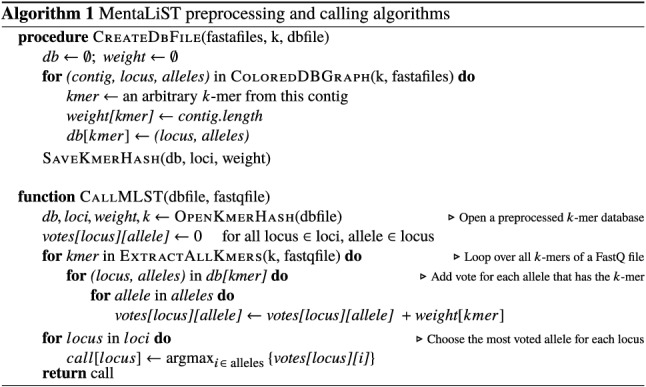
Pseudocode for the preprocessing and calling algorithms in MentaLiST.

In the current version, MentaLiST expects that all genes in the MLST scheme are present in the given sample, since it focuses on core genome MLST schemes. A pseudocode for the hash map and calling functions is shown in Fig. 2.

### Experiment design

We evaluated the performance of MentaLiST on several real and simulated datasets, measuring both the calling accuracy, the running time and the computational resources required by MentaLiST. Following a recent review of MLST tools [34], we compared MentaLiST with top-performing MLST callers, ARIBA [22], chosen for the accuracy of its calls, SRST2 (28), and stringMLST [31], which has been shown to be the fastest.

Two main datasets were used. The first dataset consists of 41 *Enterococcus faecium* samples, genotyped on a traditional MLST scheme based on seven housekeeping genes, taken from the ARIBA publication [22]. The second dataset is based on *Mycobacterium tuberculosis* samples. We created an *essential core genome MLST scheme* (ecgMLST) based on selecting a subset of 553 essential genes from the full 2891 genes in the cgMLST scheme from cgMLST.org Nomenclature Server [35], by intersecting the full cgMLST scheme gene set with the 615 essential *M. tuberculosis* genes described in Dejesus *et al*. [36]. Since the loci in this ecgMLST scheme have gene annotations that match the *M. tuberculosis* H37Rv reference genome (NCBI accession number NC_000962.3), we constructed simulated genomes by substituting the reference sequence with a randomly chosen allele in each locus position.

We also considered two large *Salmonella* MLST schemes, the SISTR scheme with 330 genes [27] and the Enterobase cgMLST scheme with 3002 genes [37]. In this case, since the annotations on both schema were not compatible with any *Salmonella* reference genome to the best of our knowledge, the simulated genomes were built by selecting a random allele for each locus and inserting random noncoding DNA from S*almonella enterica* subsp. *enterica* serovar Typhi strain CT18 (NCBI accession number NC_003198.1) between each pair of consecutive loci.

For all simulated genomes, WGS samples were created using the art read simulator [38], using the default parameters for an Illumina HiSeq 2500 machine. The samples were generated with read length and mean fragment size of 125/400 and 76/200 bp, respectively, with a 100× coverage in both cases. For the *M. tuberculosis* dataset, we also created a mixed strain dataset, with samples containing reads from two strains with varying proportions, and a coverage test dataset, where samples have coverages from 10× to 100×, in increments of 10.

The tests were run on British Columbia Genome Sciences Centre’s computer cluster using a single thread for each application. We used the default value of the parameters for all programs, except for the k-mer length for stringMLST where it was fixed to k=31 (the default is k=35), the same k value as MentaLiST.

## Results

All test results are available as a compressed file at Figshare (*Data Citation 1)*.

### *Enterococcus faecium* samples with a traditional MLST scheme

The first dataset we considered consists of 41 real *Enterococcus faecium* samples which we used to evaluate ARIBA in its original publication [22]. We used the *E. faecium* MLST scheme downloaded from PubMLST [3] containing seven housekeeping genes. This experiment provides a comparison point for the use of MentaLiST on a classical, small-scale, MLST scheme.

As expected for a traditional MLST scheme, all tested methods made identical calls on all 41 samples, except for SRST2, where on two samples the call for gene *ddl* was different from the other callers, 11 versus 5 on both cases, and had the flags ‘*?’ indicating mismatches and uncertainty due to a low depth of coverage in certain parts of the gene, according to SRST2 documentation. This is intriguing since these two alleles differ by seven base pairs at the end of the sequence, and the input samples have a very large depth of coverage.

**Fig. 3.**
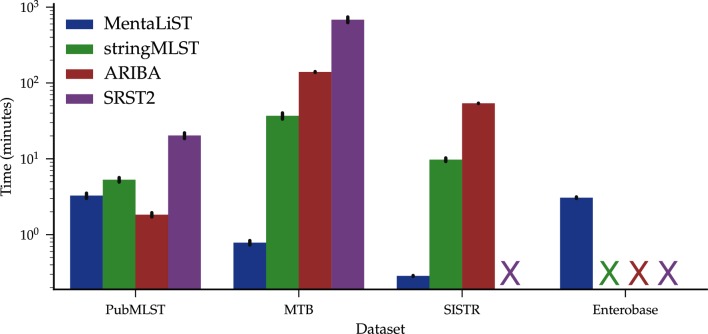
Running time for all MLST caller programs on the different schemes. X indicates that there are no results for the caller on the dataset, either because it failed or took more than 24 h. The bars represent the 95 % confidence interval.

The main difference between the callers was the runtime, as shown in Fig. 3 (PubMLST dataset). ARIBA, taking around 100 s per sample on average, was the fastest. This is the only dataset where MentaLiST was slower on average than ARIBA, due to the very high depth of coverage of the samples, most between 800× and 1200×. This indicates that ARIBA scales better than MentaLiST with the depth of coverage, but not with the number of loci in the MLST scheme, as will be shown in the results on the larger schemes.

### Essential cgMLST scheme for *Mycobacterium tuberculosis*

Our second experiment used the ecgMLST scheme for *Mycobacterium tuberculosis*, and focused on the impact of depth of coverage on the accuracy of type calls. We constructed three simulated *M. tuberculosis* datasets, and for each one we randomly selected reads to simulate a depth of coverage ranging from 10× to 100× in increments of 10×.

**Fig. 4.**
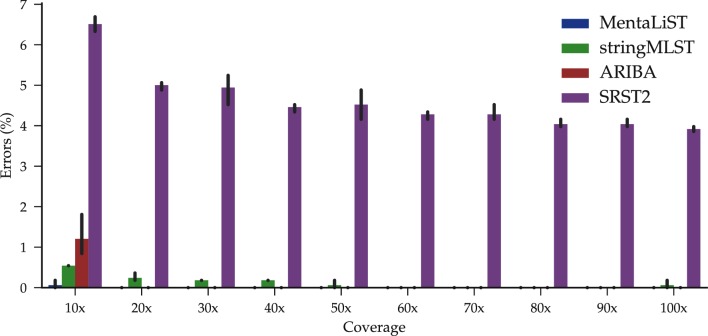
Average number of calling errors from three *M. tuberculosis* simulated samples, with varying depth of coverage and using the 553 gene ecgMLST scheme. The bars represent the 95 % confidence interval.

MentaLiST was at least two orders of magnitude faster than the other callers, as shown in Fig. 3 (MTB dataset). We see in Fig. 4 that MentaLiST and ARIBA were the only callers that accurately predicted all 553 genes at a 20× coverage, with MentaLiST also making fewer errors at a 10× coverage. With coverage larger than 60×, all callers except SRST2 are error-free.

### Mixed *M. tuberculosis* samples

Isolates from patients that suffer from an infection with more than one strain are not uncommon, with important pathological implications [39], and result in challenging WGS datasets, especially in terms of identifying the types present as well as their relative abundance [40, 41]. To assess the performance of MentaLiST in that context, we generated simulated datasets composed of two strains at various levels of relative abundance. We generated three additional random strains, as described above, and mixed them with the previously described ones to generate three samples with two strains each, and relative abundance of the major strain, denoted by *p_major_*, ranging from 50 to 95 %.

**Fig. 5.**
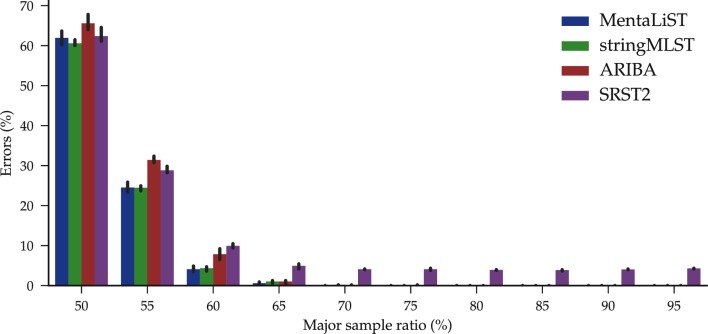
Average number of calling errors of three *M. tuberculosis* simulated datasets as a function of the proportion of the minor strain, using the 553 gene ecgMLST scheme. The bars represent the 95 % confidence interval.

The accuracy of all callers is similar, and all but SRST2 return only correct calls when *p_major_* was greater than or equal to 0.7 (see Fig. 5), showing the robustness of all methods to mixed strain data.

### Simulated *Salmonella* datasets

In the last test, we used simulated genomes of the Gram-negative bacterium *Salmonella* using two MLST schemes, the SISTR cgMLST scheme with 330 genes [27] and the Enterobase cgMLST scheme with 3002 genes [37]. We generated 10 genomes per scheme, and created two next-generation sequencing (NGS) datasets per genome with art, as described above.

Since the Enterobase scheme is a very large scheme, containing 3002 genes with up to 2000 alleles each, only MentaLiST was able to run on this scheme, with the other callers either failing to run or taking more than 24 h per sample to run, at which point we cancelled the run.

**Fig. 6.**
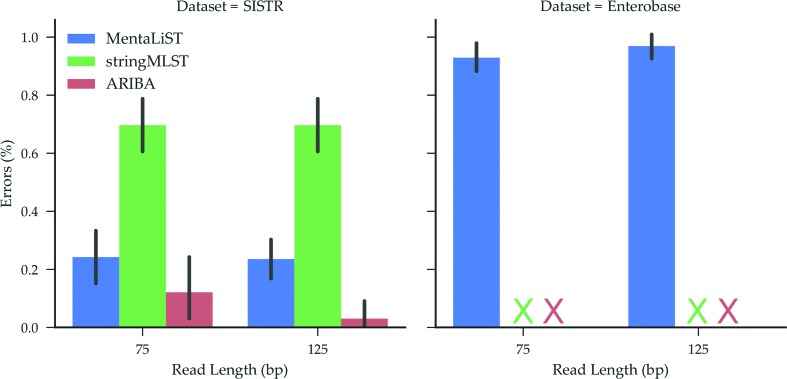
Average percentage of wrong calls on 10 simulated *Salmonella* samples with different read lengths for the SISTR (left) and Enterobase (right) cgMLST schema. X indicates that there are no results for the caller on the dataset, either because it failed or took more than 24 h. The bars represent the 95 % confidence interval.

Using the SISTR scheme, stringMLST and ARIBA took an average of 10 and 50 minutes per sample to complete, respectively. This is around two orders of magnitude slower than MentaLiST, which took 15 s per sample on average (see Fig. 3, SISTR dataset). In terms of precision, ARIBA had the best performance, especially on the 125-bp datasets, as shown in Fig. 6. For all samples but one, MentaLiST and ARIBA had either 0 or 1 error, with no errors on 80 and 25 % of the samples, respectively.

For the Enterobase scheme, on average around 28 and 29 of MentaLiST's calls per sample were wrong with read lengths of 75 and 125 base pairs, respectively, for an error rate slightly below 1 %.

## Discussion

### The importance of handling large MLST schemes

As demonstrated by our results, MentaLiST successfully handles large MLST schemes, including core genome (cgMLST) schemes, up to a few thousand genes and thousands of alleles.

It has been previously recognized that these schemes are more accurate at identifying related strains in a surveillance context [15, 18, 19] as they provide a higher resolution than the traditional schemes based on a handful of housekeeping genes.

While handling a scheme with thousands of genes and hundreds of alleles for each one is certainly taxing in terms of computational resource requirements, MentaLiST’s memory-efficient approach using the coloured de Bruijn graph to select only a subset of the k-mers and using the simple, yet effective idea of storing the complement of a list of alleles containing a given k-mer when that list contains more than half of all the alleles for a given gene, greatly reduces both the time and the memory required for allele calling, without any adverse effect on the accuracy of the calls.

In addition, thanks to an efficient implementation in the Julia language [42], MentaLiST significantly outperforms its closest competitors in terms of resource usage while providing the same or better accuracy, as we discuss in the following subsection.

### Comparison with other MLST software

Since the majority of the existing publicly available software require either a mapping of the short reads onto a reference genome or their assembly into contigs as a preprocessing step, we did not compare our performance to theirs, as the amount of time and memory they would consume would likely make their practical application nearly prohibitive on the kind of large-scale MLST schemes that we considered here. This left us with two tools in a comparable category – StrainSeeker [32] and stringMLST [31]. The former requires a guiding tree to be constructed on the alleles, and was thus excluded from the comparison. Instead, we added ARIBA [22] and SRST2 [28], which, although not necessarily designed to work on large MLST schemes, are commonly used for MLST genotyping.

**Fig. 7.**
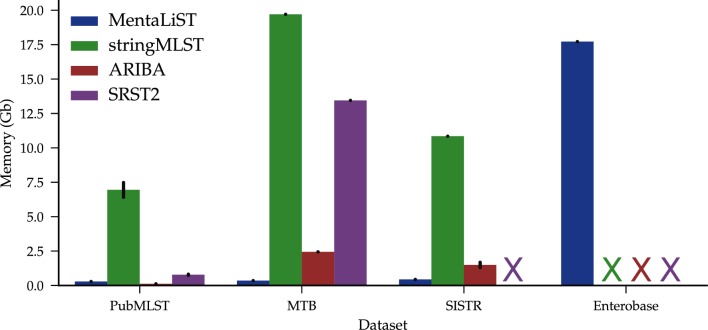
Peak memory usage for all MLST callers on the different schemes. X indicates that there are no results for the caller on the dataset, either because it failed or took more than 24 h. The bars represent the 95 % confidence interval.

Our results show that MentaLiST runs faster than stringMLST and uses less memory (Fig. 7), while consistently providing the same or better call accuracy. Furthermore, this accuracy is always the same or better than that of ARIBA except for a slight difference on the reads generated from the SISTR database. Therefore, our results enable us to confidently state that MentaLiST is at least comparable to the best-performing MLST callers in the class of the tools able to handle large MLST schemes with a reasonable amount of computational resources.

## Data bibliography

1. Feijao, P. FigShare https://doi.org/10.6084/m9.figshare.c.3856300.v2.
